# Low Ankle-Brachial Index Is Associated with Early-Stage Chronic Kidney Disease in Type 2 Diabetic Patients Independent of Albuminuria

**DOI:** 10.1371/journal.pone.0109641

**Published:** 2014-10-29

**Authors:** Xuehong Dong, Dingting Wu, Chengfang Jia, Yu Ruan, Xiaocheng Feng, Guoxing Wang, Jun Liu, Yi Shen, Hong Li, Lianxi Li

**Affiliations:** 1 Departments of Endocrinology and Metabolism, Sir Run Run Shaw Hospital, School of Medicine, Zhejiang University, Hangzhou, P. R. China; 2 Department of Epidemiology and Health Statistics School of Public Health, Zhejiang University, Hangzhou, P. R. China; 3 Department of Endocrinology and Metabolism, Shanghai Jiao Tong University Affiliated Sixth People’s Hospital, Shanghai, P. R. China; Baker IDI Heart and Diabetes Institute, Australia

## Abstract

**Aims:**

The role of low ankle-brachial index (ABI) in early-stage chronic kidney disease (CKD) is not fully known. This study was designed to investigate the prevalence of low ABI in early-stage CKD defined as an estimated glomerular filtration rate (eGFR) between 60–89 ml/min/1.73 m^2^ of type 2 diabetic patients without albuminuria and to determine the association between the low ABI and mildly decreased eGFR.

**Methods:**

The cross-sectional study enrolled 448 type 2 diabetic patients with normoalbuminuria. The patients were stratified into two groups according to the CKD-EPI eGFR level: the normal group with eGFR level ≥90 mL/min/1.73 m^2^ and the lower group with eGFR of 60–89. ABI was categorized as normal (1.0–1.39), low-normal (0.9–0.99), and low (<0.9). Both stepwise forward multiple linear regression and binary logistic regression analyses were performed to examine the association between ABI categories and eGFR levels and to assess the relation of low ABI and early-stage CKD.

**Results:**

The prevalence of low ABI in early-stage CKD of type 2 diabetic patients without albuminuria was 39.5%. Low ABI was associated with an approximate 3-fold greater risk of early-stage CKD in bivariate logistic regression analysis, and remained significantly associated with a 2.2 fold risk (95% confidence interval: 1.188–4.077; *P* = 0.012) after adjusting traditional chronic kidney disease risk factors.

**Conclusions:**

There was a high prevalence of low ABI in early-stage CKD patients of type 2 diabetes with normoalbuminuria and a close relation between low ABI and early-stage CKD, suggesting that we should pay much more attention to the patients who have only mildly decreased eGFR and normoalbuminuria but have already had a low ABI in clinic work and consider the preventive therapy in early stage.

## Introduction

The recently epidemic survey showed the prevalence of diabetes among a representative sample of Chinese adults at 11.6% and the population had been up to 113 million in 2010 [1]. Diabetes affecting the kidney, or diabetic nephropathy, affects approximately one third of patients with either type 1 or type 2 diabetes mellitus [2]. Our previous population-based study in Shanghai showed the prevalence of chronic kidney disease (CKD) and albuminuria were 32.7% and 44.2%, respectively [3], consistent with the data from Nanjing [4]. These data suggest diabetes may have reached an alert level in China with the potential for a major epidemic of diabetes-related complications, especially CKD.

Small amounts of albumin in the urine, or microalbuminuria is the current early biomarker. However, its association with progression to renal failure is unclear; as microalbuminuria does not always lead to progressive renal failure [5]. Ankle-brachial index (ABI) is a marker of generalized atherosclerosis that is associated with an increased risk of cardiovascular disease, cardiovascular mortality, and all-cause mortality [6]. In relation to kidney function, prior research indicated that low ABI (<0.9) was common in general populations with CKD [7], [8], [9] as well as in diabetic patients [10], [11]. However, some of these studies examined the relation between kidney dysfunction and ABI without adjusting for albuminuria, and most of these studies defined CKD as an estimated glomerular filtration rate (eGFR) less than 60 ml/min/1.73 m^2^ (CKD stage 3–5). While expert consensus proposes a level of <60 ml/min/1.73 m^2^ [12], two recent studies with long-term follow-up suggested that the increased cardiovascular mortality may begin even earlier, perhaps at eGFR levels below 90 ml/min/1.73 m^2^ [13], [14]. The underlying risk factors and mechanisms of early-stage CKD, especially in diabetic patients who have mildly decreased eGFR, are far from completely established.

Therefore, this study aimed first to investigate the prevalence of low ABI in early-stage CKD (defined as an eGFR between 60–89 ml/min/1.73 m^2^) of type 2 diabetic patients without albuminuria and second to determine the association between the low ABI and mildly decreased eGFR in these patients.

## Methods

### 1. Study Population

This cross-sectional study was performed in a population of 1117 patients diagnosed type 2 diabetes at the department of Endocrinology and Metabolism of the Sir Run Run Shaw hospital between January 2010 and December 2012. Among these participants, 441 patients with microalbuminuria defined as urinary albumin excretion rate 30–300 mg/24 h or macroalbuminuria defined as urinary albumin excretion rate ≥300 mg/24 h [15] and 5 patients aged≤20 years were excluded from the study. 130 patients taking either angiotensin converting enzyme inhibitors or angiotensin II receptor subtype AT-1 blockers were further excluded. 41 patients with acute infection including pneumonia, diarrhea, cholecystitis, 31 patients accepted kidney operation such as nephrectomy or getting chronic kidney disease as chronic glomerulonephritis, nephropyelitis, hydronephrosis, 12 patients at the end stage of all kinds of cancers and 9 patients whose eGFR was lower than 60 mL/min/1.73 m^2^ were also excluded. At last, 448 patients were enrolled in the study. All participants provided written informed consent, and the study was approved by the investigational review boards of the Sir Run Run Shaw Hospital of Zhejiang University.

### 2. History collection

Interviews were conducted by trained examiners who used a well-established questionnaire to collect demographic information of the study participants including date of birth, sex, smoking status, and personal medical history. Weight and height were measured while patients were dressed in light clothing. Body mass index (BMI) was calculated as weight (kg) divided by the square of the height (m). Waist circumference was measured to the nearest 0.1 cm at expiration along a horizontal plane through the abdomen at the level of the midpoint between the lowest rib and the iliac crest. Blood pressure (BP) was measured twice with the subjects in the sitting position after a 5-min rest. The lower value of two measurements was used for the study.

### 3. Laboratory Measurement

Venous blood samples and urinary specimens were collected in the morning following an overnight fast. HbA1c was examined using an automatic analyzer (VARIANT II, BIO-RAD Laboratories, Inc., California, USA). Lipid profiles including triglyceride, total cholesterol, high-density lipoprotein cholesterol, low-density lipoprotein cholesterol and very low density lipoprotein cholesterol, fasting blood glucose, fasting insulin, fasting C peptide, serum creatinine, serum uric acid and C reactive protein levels were measured using another automatic analyzer (AEROSET; Abbott Laboratories, Abbott Park, Illinois, USA).

### 4. Diagnosis of diabetic retinopathy and neuropathy

Diabetic retinopathy (DR) was assessed by the Digital non-mydriatic fundus photography (Nonmyd; Kowa Company, Ltd.; Japan) according to the protocol previously reported [16], [17]. DR was classified as none and DR containing mild non-proliferative DR, moderate non-proliferative DR, severe non-proliferative DR, proliferative DR, and diabetic macular edema by a trained ophthalmic photographer and a retinal specialist. The diagnosis of diabetic neuropathy was depending on the nerve conduction study performed by a trained physiatrist. Electrophysiological tests were done in recommended standard situations [18] for all patients by Synergy electromyograph machine (Keypoint; Dantec Dynamics A/S; Denmark), which included ulnar and median nerves (sensory and motor fibers) in upper extremities and sural (sensory), deep peroneal and tibial (motor) nerves in lower extremities. Diagnosis of diabetic neuropathy was based on at least one abnormal nerve conduction result.

### 5. Calculation of eGFR

eGFR was estimated using the CKD Epidemiology Collaboration (CKD-EPI) Study equation [19]. eGFR = ***a***×(serum creatinine/***b***)***^c^***×(0.993)^age^. The variable **a** takes on the following values on the basis of Asia race and sex: Women = 144, Men = 141. The variable **b** takes on the following values on the basis of sex: Women = 0.7, Men = 0.9. The variable **c** takes on the following values on the basis of sex and creatinine measurement: Women: Serum creatinine≤0.7 mg/dl = −0.329, Serum creatinine>0.7 mg/dl = −1.209; Men: Serum creatinine≤0.9 mg/dl = −0.411, Serum creatinine>0.9 mg/dl = −1.209. The enrolled patients were stratified into two groups according to the CKD-EPI eGFR level: the normal group with eGFR level ≥90 mL/min/1.73 m^2^ and the lower group with eGFR of 60–89 mL/min/1.73 m^2^.

### 6. ABI Measurement

The ABI measurements were performed in a supine position and BP was measured in the bilateral brachial and dorsalis pedis arteries with an 8-MHz Doppler probe (Vista AVS; Summit Doppler Systems, Inc., USA). According to the guidelines of American Heart Asso­ciation [20], ABI was calculated as the ratio of the higher value of the systolic BP of the two ankle ar­teries of that limb (either the anterior or the posterior tibial ar­tery) and the higher value of the two brachial systolic BP. For each patient, the lower ABI from both legs was used for further evaluation. Among the 448 enrolled patients, ABI levels ranged from 0.19 to 1.39. Therefore, we categorized ABI into 3 groups: normal (1.0–1.39), low-normal (0.9–0.99), and low (<0.9).

### 7. Statistical Analysis

Statistical analyses were performed using the SPSS 15.0 software package (SPSS Science, v. 15.0, Chicago, IL). The continuous variables were compared using the *t* test; categorical variables were compared using the x^2^ test; and all variables were adjusted by age and sex. Both stepwise forward multiple linear regression and binary logistic regression analyses were performed to examine the association between ABI categories and eGFR level and to assess the relation of low ABI and early-stage CKD. For all analyses, participants with eGFR of 90 mL/min/1.73 m^2^ or higher were served as the referent category with which the other groups were compared. Differences with *P*<0.05 (two-tailed) were considered statistically significant.

## Results

### Characteristics of the participants

The overall prevalence of early-stage CKD defined by CKD-EPI eGFR 60–89 ml/min per 1.73 m^2^ with normoalbuminuria was 19.2%(Table 1). Compared with participants whose eGFR levels higher than 90 mL/min/1.73 m^2^, those with eGFR 60–89 mL/min/1.73 m^2^ were older (50±11 VS 63±11 years, *P*<0.001), more frequently female, had a slightly lower prevalence of smoking habits (37%, and 14% respectively, *P* = 0.041). After adjustment for age and sex, the lower eGFR group were more likely to have a higher level of fasting C peptide (648±393 VS 801±513 pmol/l, *P* = 0.008) and a lower level of HbA1c (9.5±2.3% VS 8.8±2.5%, *P* = 0.038). The prevalence of hypertension, diabetic neuropathy and DR of the lower eGFR group were elevated and the duration of diabetes was longer, compared with the normal eGFR group. However, these differences were all attenuated and no longer significant after multivariable adjustment. There were no other significant differences between the two groups.

**Table 1:** Characteristics of the participants with normal or mildly decreased eGFR levels.

	eGFR		
	≥90 mL/min/1.73 m^2^	60–89 mL/min/1.73 m^2^	Age- and sex- adjusted *P*
n	362	86	
Age (years)	50±11	63±11	0.000
Male (%)	66.9	50.0	0.004
Duration of diabetes(years)	5±7	7±6	0.671
Hypertension (%)	23.5	50.0	0.071
The history ofsmoking (%)			
Never	53.6	72.1	
Past	8.6	13.9	0.985
Current	37.8	14.0	0.041
BMI(kg/m^2^)	24.4±4.3	23.4±3.2	0.195
Waist(cm)	87.7±10.1	86.8±10.1	0.799
systolic BP(mm/Hg)	125±17	128±18	0.355
diastolic BP(mm/Hg)	75±12	72±12	0.896
Diabetic retinopathy (%)	25.1	39.5	0.275
Diabetic neuropathy (%)	29.0	41.9	0.802
ABI categories(%)			
Normal	53.8	38.4	
Low normal	27.4	22.1	0.738
Abnormal	18.8	39.5	0.013
24 hMA(mg/24 H)	11.1±6.9	12.3±7.4	0.140
HbA1c (%)	9.5±2.3	8.8±2.5	0.038
FBG(mg/dl)	143±47	139±55	0.386
Fasting insulin(uIU/ml)	7.6±8.8	7.9±5.4	0.967
Fasting Cpeptide (pmol/l)	648±393	801±513	0.008
Triglyceride (mmol/l)	1.77±1.61	1.67±1.26	0.765
TC(mmol/l)	4.52±1.06	4.71±0.93	0.092
HDL-c (mmol/l)	1.09±0.34	1.16±0.33	0.901
LDL-c (mmol/l)	2.32±0.74	2.47±0.80	0.108
C reactive protein(mg/L)	5.5±15.9	7.6±19.4	0.855
Treatment ofdiabetes (%)			
None	29.3	25.6	
Oral Drugs	55.3	52.3	0.537
Insulin	5.8	11.6	0.357
Both	9.6	10.5	0.722

Abbreviations: BMI: body mass index; BP: blood pressure; MA: microalbminurine; FBG: fasting blood glucose; TC: total cholesterol; HDL-c: high-density lipoprotein cholesterol; LDL-c: Low-density lipoprotein cholesterol;

None: the patients never took anti-diabetic drugs. Both: the patients were taking oral drugs and subcutaneous injection of insulin at the same time.

Unless otherwise indicated, data are reported as mean ± SD. The referent category for *P* value comparisons is eGFR higher than 90 mL/min/1.73 m^2^.

Among the 448 studied participants, 22.8% (n = 102) had ABI measurements <0.90, 26.3% (n = 118) had ABI between 0.90 and 0.99, and 50.9% (n = 228) had normal ABI levels between 1.0 and 1.39, respectively. HbA1c, fasting blood glucose and lipid levels showed no differences between the three groups. Compared with the normal ABI group, the low ABI group had significantly higher systolic BP and lower waist circumference. The patients in low ABI group were older, more frequently female, had relatively higher prevalence of hypertension, lower prevalence of smoking habits and decreased eGFR level than those in normal or low-normal group (Table 2).

**Table 2:** Characteristics of the participants with different ABI categories.

ABI			
	normal (1.0–1.39)	low-normal (0.9–0.99)	low (<0.9)
n	228	118	102
Age (years)	50±12	51±12	57±12* †
Male (%)	72.4	63.6**	44.1* ††
Duration of diabetes (years)	5.0±5.0	5.9±9.8	6.1±5.6
Hypertension (%)	23.2	28.0	41.2** ††
The history of smoking (%)			
Never	51.8	56.8	69.6
Past	10.1	8.5	9.8
Current	38.2	34.7	20.6** ††
BMI(kg/m^2^)	24.6±4.2	23.7±3.3**	24.0±4.5
Waist circumference(cm)	89.3±10.4	85.6±9.8**	85.9±9.0**
Systolic BP(mm/Hg)	124±17	124±16	131±18** ††
Diastolic BP(mm/Hg)	74±12	73±12	76±11
Diabetic retinopathy (%)	26.3	30.5	28.4
Diabetic neuropathy (%)	33.8	25.4	33.3
eGFR(mL/min/1.73 m^2^)	105±15	105±17	96±16* †
24h MA(mg/24 H)	11.0±6.9	12.0±6.8	11.2±7.5
HbA1c (%)	9.5±2.3	9.4±2.4	9.1±2.2
FBG(mg/dl)	140±46	147±52	142±51
Fasting insulin (uIU/ml)	7.6±8.7	6.3±4.4	9.3±10.6††
Fasting C peptide (pmol/l)	623±370	705±463	772±466**
Triglyceride (mmol/l)	1.76±1.73	1.74±1.18	1.74±1.48
TC(mmol/l)	4.45±1.01	4.65±1.00	4.69±1.13
HDL-c (mmol/l)	1.07±0.32	1.14±0.37	1.15±0.31
LDL -c(mmol/l)	2.30±0.73	2.37±0.73	2.44±0.82
C reactive protein(mg/L)	6.2±16.8	6.7±21.0	4.4±8.8
Treatment of diabetes (%)			
None	28.9	35.6	19.6
Oral Drugs	54.8	52.5	56.9††
Insulin	7.5	4.2	8.8††
Both	8.8	7.6	14.7** ††

*P<0.05, **P<0.001 compared with group of normal ABI.

† *P*<0.05,

†† *P*<0.001 compared with group of low-normal ABI.

Abbreviations: BMI: body mass index; BP: blood pressure; MA: microalbminurine; FBG: fasting blood glucose; TC: total cholesterol; HDL-c: high-density lipoprotein cholesterol; LDL-c: Low-density lipoprotein cholesterol.

### Percentage of ABI categories in different eGFR groups

**Figure 1:**
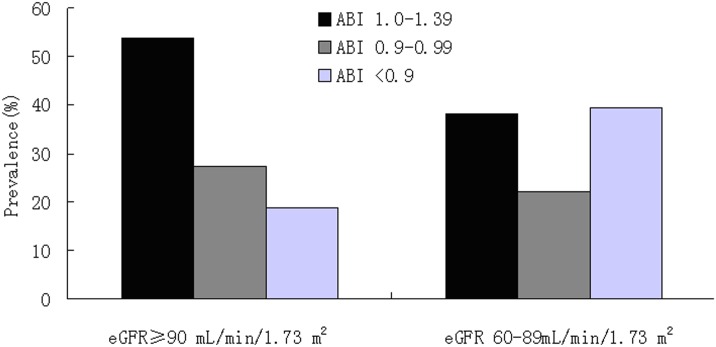
The distribution of ABI categories in two groups according to the eGFR level.

**Figure 2:**
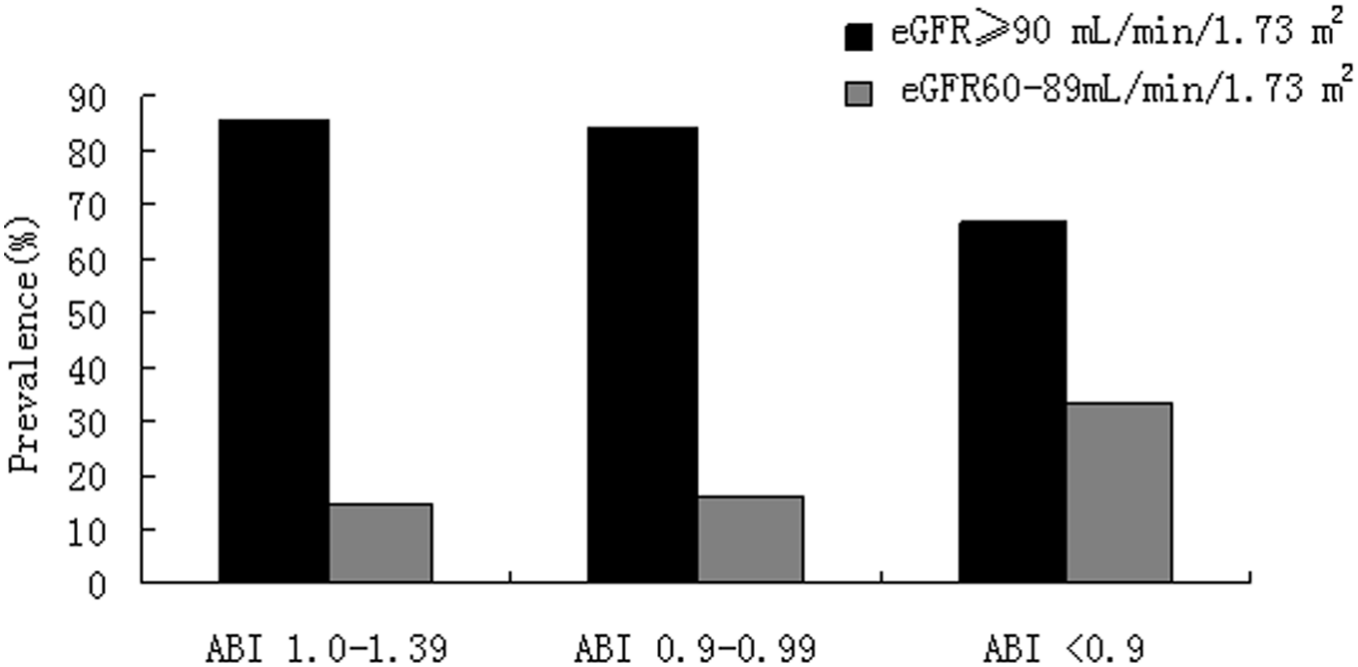
Proportion of the sample that developed the kidney function decline by ABI categories.

The prevalence of low ABI in early-stage CKD (CKD stage 2) of type 2 diabetic patients without albuminuria was 39.5%. Meanwhile, the prevalence of low ABI in normal eGFR group was only 18.8%. Normal ABI was significantly more frequent in the group showing normal eGFR levels than in those with CKD stage 2 (Figure 1). On the other side, Figure 2 showed a higher proportion of participants with early-stage CKD in the groups with low ABI than those with low-normal or normal ABI.

### Relation between low ABI and eGFR in early-stage CKD

Low ABI was associated with an approximate 3-fold greater risk of eGFR lower than 90 in bivariate logistic regression analysis. The association of ABI <0.9 with early-stage CKD was moderately attenuated in multivariable logistic regression models, but low ABI remained significantly associated with a 2.2 fold risk (95% confidence interval [CI] 1.188–4.077; *P* = 0.012) for mildly decreased eGFR (Table 3). Furthermore, we evaluated the association of eGFR as a continuous measure with different ABI categories and found a significant relation (β = –0.100; *P* = 0.043) after adjustment for age, sex, the history of hypertension, the duration of diabetes, complications including diabetic neuropathy and retinopathy, smoking status, BMI, triglyceride and C reactive protein levels.

**Table 3:** Association of Ankle-Brachial Index(ABI) with early-stage CKD.

ABI	Bivariate OR(CI)	*P* Value	Multivariate* OR(95% CI)	*P* Value
1.0–1.39	1.0(referent)	referent	1.0(referent)	referent
0.9–0.99	1.134(0.614–2.096)	0.688	1.269(0.65–2.477)	0.485
<0.9	2.955(1.700–5.135)	0.000	2.201(1.188–4.077)	0.012
*P* #value for trend		0.000		0.043

Abbreviations: CI, confidence interval; OR, odds ratio.

*The following covariates ascertained at baseline were included in multivariate analysis: age, sex, the history of hypertension, the duration of diabetes, smoking status, complications including diabetic neuropathy and retinopathy, body mass index, HbA1c, HDL and LDL cholesterol values, triglyceride levels.

# The following covariates ascertained at baseline were included in regression analysis: age, sex, the history of hypertension, the duration of diabetes, smoking status, complications including diabetic neuropathy and retinopathy, BMI, triglyceride and C reactive protein levels.

## Discussion

A main finding of this study was the alarming high prevalence of low ABI (nearly 40%) in early-stage CKD (CKD stage 2) of type 2 diabetic patients without albuminuria. Another important finding of this study was that low ABI was significantly associated with mildly decreased eGFR after adjusting traditional chronic kidney disease risk factors. To the best of our knowledge, this is the first study to focus on the normoalbuminuric early-stage CKD and to evaluate the precise role of low ABI in eGFR declining in Chinese patients with type 2 diabetes.

The association of low ABI with cardiovascular disease has been well established in a number of populations, including diabetic patients, hypertensive patients and general subjects [21], [22], [23], but not until recently have several studies reported the close relation of low ABI and CKD in general population. The previous cross-sectional studies showed that low ABI was associated with ∼50% higher odds of having eGFR <90 mL/min/1.73 m^2^ compared with ABI of 1.00–1.19 [24] as well as with elevated serum creatinine level [25]. In the 3 years’ ARIC study, participants with an ABI lower than 0.9 had more than 4-fold odds of experiencing a 50% rise in creatinine level compared with those with an ABI of 1 or higher, and the association persisted after adjustment for known predictors of renal functional decline [7]. The Framingham Offspring 10 year’s follow-up Study showed low ABI was associated with 5.73-fold increased odds of rapid eGFR decline and a 2.51-fold increased odds of stage 3 CKD [26]. Our findings were consistent with the current body of literature and further indicated that type 2 diabetic patients with normoalbuminuria in early-stage CKD (CKD stage 2) had a ∼40% of low ABI prevalence, suggesting the importance to explore the relation of low ABI and eGFR in normoalbuminuric diabetic patients.

In the DEMAND study, 20.5% of 11,315 subjects with reported decreased kidney function were found to be normoalbuminuric [27]. In the NEFRON survey, more than half (55%) of all diabetic patients with an eGFR <60 ml/min per 1.73 m2 had normoalbuminuria and most (98%) of them were also reported as being persistently normoalbuminuric [28]. For healthy nondiabetic individuals, the rate of decline in GFR with age has been reported to range between 0.6 and 1.0 mL/min/1.73 m^2^·year–^1^ when estimated from serum creatinine or creatinine clearance [29], [30], [31]. However, Richard J. Macisaac et al showed that the rate of decline in renal function for normoalbuminuric patients (–4.6±1.0 mL/min/1.73 m^2^·year–^1^) was clearly greater than that related to aging alone and was not different to that observed for micro- and macroalbuminuric (–2.8±1.0, and −3.0±0.7 mL/min/1.73 m^2^·year–^1^) patients [32]. Similar to our previous and other studies [3], [4], [33], we found that 60.5% of the overall 1117 type 2 diabetic participants were normoalbuminuric and, among them the prevalence of early-stage CKD defined by CKD-EPI eGFR 60–89 ml/min per 1.73 m^2^ was 19.2%. Low ABI was associated with an approximate 3-fold greater risk of eGFR lower than 90 in bivariate logistic regression analysis, and remained significantly associated with a 2.2 fold risk for early-stage CKD after adjusting traditional chronic kidney disease risk factors. Together these findings suggest that patients with type 2 diabetes can commonly progress to a significant degree of renal impairment while remaining normoalbuminuric. Our findings further support the hypothesis that the renal manifestation of systemic arteriosclerosis can be exist in a very early stage of CKD.

In a recent study, typical glomerular changes of diabetic nephropathy were observed in 22 of 23 subjects (mean eGFR 31 mL/min/1.73 m^2^) with micro- or macroalbuminuria compared with 3 of 8 subjects with normoalbuminuria [34]. By contrast, predominantly interstitial or vascular changes were seen in only 1 of 23 subjects with micro- or macroalbuminuria compared with 3 of 8 normoalbuminuric subjects. Varying degrees of arteriosclerosis were seen in seven of eight subjects with normoalbuminuria. Consistent with the previous data [10], [35], our study demonstrated that the abnormal ABI, but not other diabetic microvascular disease such as diabetic retinopathy and neuropathy, was significantly associated with mildly decreased eGFR after adjusting for other risk factors. Further studies will be needed to clarify the initial renal structural changes in early-stage CKD and explore the exact role of atherosclerosis in CKD without normoalbuminuria. An important methodological strength of the current study, as opposed to other previous population-based studies, was concerning and therefore excluding the patients with medications of the renin-angiotensin-aldosterone system inhibitors which have been demonstrated by multiple trials that can decrease proteinuria, preserve renal function, hence confuse the results [36], [37], [38]. Another strength of the study was that we determined the entire spectrum of patients in early-stage CKD independent of albuminuria, who were easily to be ignored in clinical work but deserved to be pay more attention to by physicians. The main limitation was the cross-sectional design that did not allow us to examine the effect of low ABI on the development of CKD. Thus, prospective studies of a larger sample should be conducted and the primary outcome of end stage renal disease incidence should be used. Other possible limitations included the incomplete records of hypoglycemia, and the hospital-based study cohort making selection bias a potential confounding factor. However, the imbalance of HbA1c levels between the two groups can also be explained by the fact that patients with different eGFR levels had diverse therapeutic regimen.

In summary, the prevalence and the important role of low ABI in early-stage CKD patients of type 2 diabetes who have normoalbuminuria have been well defined in our study. The results imply that low ABI level contributes to the risk of various degrees of renal atherosclerosis, and that we should pay much more attention to the patients who have only mildly decreased eGFR and normoalbuminuria but have already had a low ABI in clinic work and consider the preventive therapy in early stage of CKD.
